# Changes in children’s television and computer time according to parental education, parental income and ethnicity: A 6-year longitudinal EYHS study

**DOI:** 10.1371/journal.pone.0203592

**Published:** 2018-09-07

**Authors:** Marieke De Craemer, Maïté Verloigne, Ariane Ghekiere, Anne Loyen, Patricia Dargent-Molina, Johannes Brug, Nanna Lien, Karsten Froberg, Niels Wedderkopp, Sebastien Chastin, Greet Cardon, Jelle Van Cauwenberg

**Affiliations:** 1 Department of Movement and Sport Sciences, Ghent University, Ghent, Belgium; 2 Research Foundation Flanders, Brussels, Belgium; 3 Department of Public Health, Ghent University, Ghent, Belgium; 4 Department of Public and Occupational Health, VU University Medical Center, Amsterdam, The Netherlands; 5 Early Determinants of Children’s health and Development Team (ORCHAD), Inserm Epidemiology and Biostatistics Sorbonne Paris Cité Center (CRESS), Villejuif, Paris Descartes University, Paris, France; 6 Department of Epidemiology and Biostatistics, VU University Medical Center, Amsterdam, the Netherlands; 7 Amsterdam School for Communication Research, University of Amsterdam, Amsterdam, the Netherlands; 8 Department of Nutrition, Institute of Basic Medical Sciences, Oslo, Norway; 9 Department of Exercise Epidemiology, University of Southern Denmark, Odense, Denmark; 10 Department of Regional Health Research, University of Southern Denmark, Odense, Denmark; 11 School of Health and Life Sciences, Glasgow Caledonian University, Glasgow, the United Kingdom; Erasmus Graduate School of Social Science and the Humanities, NETHERLANDS

## Abstract

**Objectives:**

To investigate changes in children’s television and computer time according to three socio-economic status (SES) indicators.

**Design:**

Prospective cohort study.

**Methods:**

Data were drawn from the European Youth Heart Study and included longitudinal data collected in 1997 and 2003 in Denmark. Television and computer time were self-reported by children. Parental education, income and ethnicity were parent-reported. Baseline data were available for 549 children (47.0% boys, 9.6 years). Generalized linear mixed models analyzed whether changes in television and computer time from baseline to follow-up differed according to the SES-indicators.

**Result:**

TV viewing time increased with 25% over time (ExpB = 1.25, 95% CI = 1.04–1.50). At both time points, children with two higher educated parents viewed 25% less hours of television than children with no higher educated parents (ExpB = 0.75, 95% CI = 0.60–0.94) and one higher educated parent (ExpB = 0.75, 95%CI = 0.59–0.97). Among children with no higher educated parents the odds of being in a higher category of computer time increased with 80% over time (OR = 1.80, 95% CI = 1.24–2.60). Among children with two higher educated parents the odds of being in a higher category of computer time decreased with 45% over time (OR = 0.55, 95% CI = 0.32–0.94). The association with ethnicity showed that white children had 42% lower odds (OR = 0.58; 95% CI = 0.34–1.00) of being in a higher category of computer time than non-white children. No significant associations were found for parental income.

**Conclusions:**

The most important SES measure of screen-based behaviors in children was parental education. Ethnicity was only associated with computer time. Financial resources were less relevant for changes in television viewing and computer use.

## Background

Sedentary behavior is the collective name of a cluster of activities that are done while sitting or lying down, and which are characterized by a low energy expenditure (≤1.5 metabolic equivalents) [1]. Although more evidence exists for an association between sedentary behavior and health indicators in adults [2–5], the body of evidence in children has grown during the last years and showed an adverse association between sedentary behavior in children and adolescents and obesity, blood pressure, total cholesterol, self-esteem, social behavior problems, physical fitness and academic achievement [2, 6]. Sedentary behavior is already highly prevalent in youth, especially screen time behavior [7], for which the most recent Canadian screen time guidelines state to limit screen time behavior to a maximum of two hours per day in children and youth [8]. In addition, the American Academy of Pediatrics also recommended to limit screen time to two hours per day in 2001 and 2011, but they recently changed their recommendations—due to screens and social media being ubiquitous—into the encouragement of parents to develop a family media use plan which is specific for each family and each family member [9]. Higher screen time in children has been associated with health indicators such as unfavorable body composition, higher clustered cardio metabolic risk scores, unfavorable behavioral conduct/prosocial behavior and lower self-esteem [10]. Recent studies showed low compliance with screen time guidelines [11, 12]. The study of Hardy et al. (2017) showed that only 18% of 5- to 16-year old Australian children complied with the screen time guidelines [11]. In addition, a study with 9- to 11-year-old children comparing compliance with screen time guidelines across ten different countries showed an overall compliance of 39.3% [12]. This necessitates the development of effective interventions aiming to decrease screen time in this age group. Moreover, establishing healthy behaviors early in life might lead to sustained habits later on and in adulthood.

To inform the development of these interventions, it is important to identify specific at-risk groups with regard to sedentary behavior. In this respect, the relationship between socio-economic status (SES) and sedentary behavior has already been investigated in several studies [13–21]. Research often suggests an inverse relationship between SES and sedentary behavior [13, 14, 22, 23], which means that a higher SES is associated with less screen time. This relationship may be more complex as different indicators of SES have different associations with sedentary behavior [15]. For example, within low-middle-income countries sedentary behavior more strongly associates with income than with parental education [15]. Another study found that children from parents with a higher educational level and from less deprived areas showed more objectively measured sedentary time, although living in a household with a private garden was associated with less sedentary time [16]. In addition, the association with SES can vary between different sedentary behavior domains [15]. A lower SES, based on a composite score including household income, area deprivation and social class, was associated with more television (TV) time, but with less total sedentary time and other sitting activities during leisure time in English children and adolescents [17]. On the contrary, a higher household income was associated with more TV viewing, but with less computer use among 12- to 17-year-old US adolescents [18]. This complex association between different SES-indicators such as parental education and income and different sedentary behaviors such as TV viewing and computer use, has not been investigated longitudinally. In addition, there are other social categories such as race and ethnicity that are related to SES-indicators which could make the association with sedentary behaviors even more complex. For example, ethnic minorities generally have lower educational and income levels and live in more deprived neighborhoods [24]. It could thus be interesting to investigate the association with child ethnicity as well. Understanding which SES-indicator is most strongly associated with screen time in children could heighten comparability across studies, and could inform future studies targeting screen time in children and families from lower and higher SES as intervention strategies might differ.

Therefore, the aim of the current study was to investigate changes in TV and computer time according to three different SES measures (parental education, parental income and child ethnicity) in a 6-year longitudinal study among Danish children aged nine years at baseline. This period represents the transition from childhood to adolescence which has been associated with an increase in screen time [19], and recent studies showed low compliance rates with screen time guidelines in this age group [11, 12]. Because of the inconsistent associations between SES-indicators and screen time observed in previous studies, we were not able to postulate specific hypotheses about these differences according to SES-indicators [15–18].

## Methods

This secondary data analysis was conducted within the DEDIPAC (DEterminants of DIet and Physical ACtivity) study, which aimed to improve the understanding of the determinants of dietary, physical activity and SBs [25]. To investigate appropriate datasets to answer the research question, we used the DEDIPAC compendium, which is a systematic inventory of 114 available European datasets (unpublished data). The only dataset that could answer our research question were the data from the European Youth Heart Study (EYHS). The aim of EYHS was “to establish the nature, strength and interactions between personal, environmental and lifestyle influences on cardiovascular disease risk factors in European children” [26]. The EYHS was designed as a cross-sectional survey of children conducted in four countries: Denmark, Estonia, Norway, and Portugal [26]. In a second phase of the study, six-year follow-up data were collected, incorporating repeated measurements of the included children. As described in the protocol paper of Riddoch et al. (2005), a sampling frame of schools was compiled with the use of official lists. Schools were then stratified by sociodemographic characteristics. A minimum of 20 schools were randomly selected by using probability proportional to school size. With use of random number tables, nine-year-old children were then sampled within the different schools using the school register [26]. The present study only used the longitudinal data collected in 1997 and 2003 in 25 Danish schools which included 590 and 383 children, respectively at baseline and follow-up. Written informed consent was obtained from a parent or guardian, and the study procedures were explained verbally to all the children. Ethical approval for the study was obtained from the local research ethics committee (Research Ethics Committee of the Region of Southern Denmark—Odense University Hospital).

All child-specific variables were child-reported in a child questionnaire, and all parent-related variables were parent-reported in a parental questionnaire. Response rate at baseline was 98% [26].

### Weekday TV time

Children were asked how many hours/day they watch TV before and after school. For hours/day watching TV before school, response categories were ‘none’, ‘less than 1 hour’, ‘1–2 hours’, and ‘more than 2 hours’. For hours/day watching TV after school, response categories were ‘none’, ‘less than 1 hour’, ‘1–2 hours’, ‘2–3 hours’, and ‘more than 3 hours’. Variables were recoded into a quantitative variable (average minutes/day) using the midpoint method (e.g., ‘1–2 hours’ = 90 minutes, ‘2–3 hours’ = 150 minutes, ‘more than 3 hours’ = 210 minutes), and were subsequently summed to obtain daily TV time.

### Average daily computer time

Children were asked how many hours/day (whole day, including week and weekend days) they spent playing games or using a computer. Response categories were ‘none’, ‘less than 1 hour’, ‘1–2 hours’, and ‘more than 2 hours’. At baseline, no participants reported computer times higher than 2 hours resulting in only three different responses.

To obtain a variable representing compliance with screen time guidelines [8], we created a continuous variable for average daily computer time using the midpoint method, which was then summed with the variable daily TV time. The obtained variable represented overall daily screen time and was dichotomized into not exceeding the guidelines (≤ two hours/day, coded 0) and exceeding the guidelines (> two hours/day, coded 1).

### Ethnicity

Parents reported ethnicity, which was assessed by using five response categories (‘White’, ‘Black’, ‘Pakistan/Indian’, ‘other Asian countries’, and ‘other countries’) and were recoded into two categories, namely ‘White’ and ‘other’.

### Parental education level

Mother’s and father’s educational level were assessed at baseline. Response categories were on an 11-point scale (‘primary school (1)’, ‘secondary school (2)’, ‘EFG basic (3)’ (i.e., basic vocational education), ‘semi-skilled worker (4)’, ‘EFG 2. Part (5)’ (i.e., 2^nd^ part of basic vocational education), ‘apprentice (6)’, ‘under education (7)’, ‘basic exam (8)’, ‘short further education (9)’, ‘medium further education (10)’, and ‘long further education (11)’) and were recoded into ‘lower education’ (categories 1–8) and ‘higher education’ (categories 9–11). These two variables (one for the mother and one for the father) were then combined, so a new variable was created with three categories; both parents have a higher education (= 2), one of both parents have a higher education (= 1), and both parents have no higher education (= 0).

### Parental income

Baseline, maternal and paternal incomes were assessed using two questions with each eight response categories. These eight response categories reflected the annual income categories in absolute Danish kroner. The lowest response reflects the lowest income and the highest response reflects the highest income. Parents were asked to place themselves in one of these eight categories according to their gross income. Maternal and paternal income were averaged and used as one continuous variable [27, 28].

### Demographic variables

Children’s gender and age at baseline were obtained via the child questionnaire.

Analyses were performed in R version 3.1.1. Statistical significance was defined at p<0.05 and a trend for statistical significance at p<0.10.

Four children had no TV or computer time data at baseline and follow-up yielding an analytic sample of 586 participants. From the 586 children, 211 had missing TV and computer time data at baseline or follow-up; eight participants missed baseline measurements and 203 participants dropped-out of the study. All participants with missing data missed both TV and computer time. Participants with complete versus incomplete data were compared on (1) sex, education, ethnicity and baseline computer use by means of a chi^2^-test, (2) baseline age, BMI (based on self-reported height and weight) and income by means of an independent sample t-test and (3) baseline TV-viewing by means of a Mann-Whitney U test (since TV-viewing was positively skewed).

Our data had the following three-level hierarchical structure: repeated measures clustered within individuals and individuals clustered within schools. To take this structure into account, generalized linear mixed models (glmm) were used. Treating the repeated measures as clustered within participants has the advantage that all participants were retained in the analysis even if they missed a baseline or follow-up measurement of TV viewing/computer time (i.e., all available information is used). This is the recommended approach to analyze longitudinal data with missingness and implies that missingness can be ignored if all variables related to drop-out are included in the model [29]. The outcome ‘TV viewing’ was positively skewed and, therefore, a glmm with a gamma variance and log link function was fitted using the lme4-package [30]. Based on Akaike’s Information Criterion such a model was preferred over a general linear mixed model (i.e. a Gaussian variance and identity link function). Exponentiated coefficients from a gamma model with log link function can be interpreted as proportional differences in TV viewing with a one-unit change in the independent variable. The outcome ‘computer time’ was an ordinal variable, therefore, an ordinal logistic regression mixed model was applied using the ordinal-package [31]. The resulting odds ratios represent the odds of belonging to a higher category of computer time with a one-unit change in the independent variable. For the dichotomous compliance with screen time guidelines variable, binomial logistic regression mixed models were applied.

For each outcome variable, four steps were applied to obtain a final model. First, a basic model was fitted including one of the three SES-indicators (parental education level, parental income, ethnicity), time, sex and age (three models per outcome). In a second step, all three SES-indicators were entered simultaneously into this basic model (one model per outcome). The estimates for the SES-indicators from these two steps can be interpreted as differences in the outcome after adjustment for time. In a third step, the interaction effects between time and one of the three SES-indicators were added separately to the model fitted in the second step (three models per outcome). A significant interaction effect indicated that the change in TV viewing or computer time or odds of excessive screen time differed according to SES-indicator. Fourth, to assess whether the observed changes according to educational level were independent of income (and vice versa), a model was fitted including the three-way interaction effect between time, education and income. Given the small number of non-white participants, we could not examine a three-way interaction effect including ethnicity.

## Results

Data were collected among 590 and 383 children at baseline and follow-up, respectively. Four children had no TV or computer time data at baseline and follow-up and 37 children had no information on one or more SES measures, yielding an analytic sample of 549 participants. From these 549 participants, 193 (35.2%) had missing TV and computer time data at baseline or follow-up. From these 193, eight participants missed baseline measurements and 185 participants missed follow-up measurements (dropped-out of the study). All participants with missing data missed both TV and computer time data. This implies that all statistical models included 905 ((549*2)-193) observations of TV/computer time obtained from 549 participants. Participants with complete versus incomplete TV/computer time data did not differ in age, sex, BMI, ethnicity, education and baseline TV and computer time. Those with incomplete TV/computer time data were from families with a significantly lower parental income (3.6±1.5) than those with complete data (4.1±1.4, t = -3.6, df = 547, p<0.001).

Descriptive characteristics of the final analytic sample are shown in Table 1. At baseline, the 549 children (47.0% boys) had a mean age of 9.6±0.4 years. Most children were white (93.4%).

**Table 1:** Demographic characteristics of the sample (Baseline information, n = 549).

Sex (%boys)	47.0	
Age (M±SD)	9.6 ± 0.4	
BMI (M±SD, kg/m^2^)	17.3 ± 2.5	
Ethnicity (%non-white)	6.6	
Parental education (%)		
No parent with higher education	48.5	
One parent with higher education	27.5	
Two parents with higher education	24.0	
Parental income (M±SD)	3.9 ±1.4	
**Screen time behaviors**	**1997 (n = 541)**	**2003 (n = 364)**
Television viewing school day (min/day; Med, Q1-Q3)	60.0, 30–120	90.0, 30–150
Computer time (%)		
No computer time	30.8	43.6
Less than one hour	52.1	23.0
One to two hours	17.1	19.6
More than two hours	0.0	13.8
Compliance with screen time guidelines (% exceeding 2 hrs/day)	30.5	47.8

The amount of TV viewing increased with 25% over time, which was found to be statistically significant (ExpB = 1.25, 95%CI = 1.04–1.50) (see Table 2). In the basic model, a significant relationship for educational level was observed. Children with two higher educated parents viewed 25% less television than children with no higher educated parent (ExpB = 0.75, 95%CI = 0.60–0.94) and one higher educated parent (ExpB = 0.75, 95%CI = 0.59–0.97). These relationships remained borderline significant (p = 0.05) in the model adjusted for the other SES-indicators. Ethnicity and parental income were not significantly related to TV viewing nor were there any significant interaction effects between the SES-indicators and time. The three-way interaction effect between time, education and income was also non-significant. These findings imply that TV-viewing increased over time independently of SES, but TV viewing was lower across the two time points among children with two compared to no higher educated parents.

**Table 2:** Evolution in TV viewing or computer time according to educational level, ethnicity and parental income.

	TV viewing		Computer time		Compliance with screen time guidelines	
	Basic model^1^ ExpB (95% CI)	Adjusted for other SES-indicators^2^ ExpB (95% CI)	Basic model^1^ OR (95% CI)	Adjusted for other SES-indicators^2^ OR (95% CI)	Basic model^1^	Adjusted for other SES-indicators^2^
Time (ref. = 1997)	/^3^	1.25 (1.04–1.50)*	/^3^	1.13 (0.87–1.47)	/^3^	2.36 (1.76–3.18)*
Educational level (ref. = no parent with higher education)						
One parent with higher education	1.00 (0.80–1.24)	1.01 (0.81–1.26)	1.01 (0.75–1.36)	0.96 (0.70–1.31)	1.01 (0.72–1.43)	0.98 (0.69–1.40)
Two parents with higher education^4^	0.75 (0.60–0.94)*	0.77 (0.59–1.00) ^†^	0.92 (0.67–1.27)	0.85 (0.59–1.23)	0.46 (0.31–0.68)*	0.43 (0.27–0.68)*
Ethnicity (ref. = non-white)	0.88 (0.61–1.27)	0.92 (0.63–1.37)	0.62 (0.37–1.04)*	0.58 (0.34–1.00)*	0.75 (0.41–1.38)	0.79 (0.42–1.49)
Parental income	0.95 (0.89–1.02) ^†^	0.99 (0.92–1.07)	1.01 (0.92–1.11)	1.06 (0.95–1.18)	0.91 (0.82–1.02)	1.04 (0.91–1.18)

* p< 0.05;

^†^ p< 0.10

All models included 905 observations obtained from 549 participants in 25 schools.

ExpB = exponentiated regression coefficient of a generalized linear mixed model with gamma variance and log link function, represents the proportional difference in TV viewing with a one-unit change in the independent variable.

OR = odds ratio of an ordinal logistic mixed model, represents the odds of belonging to a higher category of computer time with a one-unit change in the independent variable.

CI = confidence interval

ref. = reference category

^1^ Adjusted for gender and age (step 1).

^2^ Adjusted for the two other SES-indicators, gender and age (step 2).

^3^ Estimates for time differed slightly between the three basic models (one for each SES indicator) and, therefore, no estimate was presented. The relationship of time was derived from the model adjusted for all SES indicators.

^4^ Compared to participants with one parent with higher education, those with two parents with higher education viewed 25% less television (Exp B = 0.75, 95%CI = 0.59–0.97). Compared to participants with one parent with higher education, those with two parents with higher education had 55% lower odds of exceeding screen time guidelines (OR = 0.45, 95%CI = 0.29–0.69).

In the main effect models (step 1 and 2) computer time did not significantly change over time (OR = 1.13, 95%CI = 0.87–1.47). Furthermore, a significant relationship with ethnicity was observed; white children had 42% lower odds (OR = 0.58, 95%CI = 0.34–1.00) of being in a higher category of computer time than non-white children. No significant relationships with educational level and parental income were observed in step 1 and 2. However, a significant interaction effect between time and educational level was observed in step 3, but the three-way interaction effect between time, education and income was non-significant. The change in computer time among children with no higher educated parents was significantly different than the change among children with one or two higher educated parents (interaction effects: OR = 0.52, 95%CI = 0.28–0.97 and OR = 0.31, 95%CI = 0.16–0.59, respectively). Among children with no higher educated parents the odds of being in a higher category of computer time increased with 80% over time (OR = 1.80, 95%CI = 1.24–2.60). Among children with one higher educated parent no changes in computer time were observed over time (OR = 0.94, 95%CI = 0.57–1.54). Among children with two higher educated parents the odds of being in a higher category of computer time decreased with 45% over time (OR = 0.55, 95%CI = 0.32–0.94).

The odds of exceeding the screen time guidelines increased significantly with 136% over time (OR = 2.36, 95%CI = 1.76–3.18) (see Table 2). A significant relationship for educational level was observed in the basic and the adjusted model. Children with two higher educated parents had 54% and 55% lower odds of exceeding the screen time guideline in comparison to children with no higher educated parent (OR = 0.46, 95%CI = 0.31–0.68) and one higher educated parent (OR = 0.45, 95%CI = 0.29–0.69). These relationships remained significant (p = 0.05) in the model adjusted for the other SES-indicators. Ethnicity and parental income were not significantly related to the odds of exceeding screen time guidelines nor were there any significant interaction effects between the SES-indicators and time. The three-way interaction effect between time, education and income was also non-significant. These findings imply that the odds of increasing the screen time guidelines increased over time independently of SES, but the odds for excessive screen time were lower across the two time points among children with two higher educated parents.

## Discussion

Overall, the results indicate that SES was inversely related to screen-based activities in children, which means that a higher SES was associated with less time spent in screen-based activities. More specifically, parental education seemed to be the most influential SES-indicator with regard to children’s sedentary activities. At both time points, TV viewing and compliance with screen time guidelines were higher in children with no higher educated parents compared to children with two higher educated parents. This is in line with the results of the longitudinal study of Brodersen et al., although another SES-indicator (neighborhood SES) and only total screen time was used [19]. For computer time, no differences between the three categories of parental education were found at baseline but the changes over time were significantly different with a less favorable change for children with no higher educated parents. A possible explanation might be that higher educated parents expose their children to technology in a different way. For example, it might be possible that parents from educated backgrounds set limits when children watch TV or use the computer. Although children with no higher educated parents were in the least favorable position for TV viewing, computer time, as well as adoption of compliance with screen time guidelines, the different change over time shows that it is important to investigate changes in TV viewing, computer time, and compliance with screen time guidelines separately [15, 17, 18]. Indeed, the educational differences in TV viewing and compliance with screen time guidelines were already apparent at age nine whereas the differences in computer time occurred later. This suggests that intervening at different stages of childhood might be relevant in order to decrease specific screen time behaviors. Future research should specifically investigate the crucial moment at which to intervene in order to get the most optimal results for the different sedentary activities. Additionally, as media opportunities dramatically changed during the last decades, it should be investigated whether the current findings are still applicable for today’s media environment.

A possible mechanism for the inverse relationship between parental education and TV viewing is that a lower parental education is associated with lower parental modelling (i.e., showing a good example), less parental co-viewing, more chance to have a TV in the bedroom and to eat meals in front of the TV [32]. Furthermore, research showed that children’s screen time is influenced by parents’ parenting practices, i.e., practices to positively influence the child’s behavior (e.g., having parental rules), and that these parenting practices tend to differ according to SES [33]. It might be possible that parenting practices mediate the relationship between (family) SES and children’s screen time, but up to now only few mediation studies have been conducted [34]. Therefore, future studies should consider the mediating effect of parenting practices on the association between SES and children’s screen time. In addition, factors such as parental modelling, parental co-viewing, the presence of a TV in the bedroom and eating meals in front of TV are of interest to be investigated in future longitudinal studies within this age group, looking at their mediating role in the association between SES and children’s screen time. In addition, it seems that the inverse association is only present (for TV viewing) or strongest (for computer use) when both parents have a higher education, which suggests that both parents’ educational level are important. For computer time, future studies should also investigate the specific mediators of the association between parental educational level and increases in computer time from childhood to adolescence.

Parental income did not significantly associate with TV viewing, computer time nor compliance with screen time guidelines. This might suggest that resources to buy screen-based devices are less relevant for sedentary activities. In low-income countries on the contrary, parental income seemed to be more influential than parental education [19]. These observed differences in associations between low- and high income countries (such as Denmark) might be explained by the fact that ownership of electronic devices and televisions are more determined by financial resources in low-income countries, which in turn could influence children’s screen time behavior. It should be noted that Denmark has a specific context which means that it performs well in many measures of well-being (e.g., work-life balance, social connections, environmental quality, civic engagement, education and skills, jobs and earnings, subjective well-being and personal security) compared to other countries [35]. Therefore, results from this study should be interpreted carefully.

We also found an association between ethnicity and computer time with non-white children having higher levels of computer time at baseline compared to white children. However, it must be acknowledged that ethnicity was roughly categorized into white and non-white children which means that it was not possible to distinguish between different specific ethnic groups. In addition, the current sample was highly skewed with the majority of children being white and only 7% of children being classified as non-white. Moreover, other studies have assessed ethnicity differently (e.g., ancestry, race), which makes it difficult to compare results across studies. However, our results support previous studies showing that non-white children have less favorable levels of sedentary behavior [19, 36, 37], which might be related to the fact that on average, European ethnic minorities live more often in lower SES neighborhoods, are lower educated and have lower income levels [24].

An important study strength is the longitudinal design in a relatively large sample which enabled us to investigate how sedentary behaviors changed over the years according to SES. Secondly, we used three different SES-indicators and two screen-based outcomes. However, to acquire in-depth insights into the association between SES and sedentary behavior, other indicators such as neighborhood SES and outcomes such as objectively measured sedentary behavior could also be included in future studies. There are also some limitations. First, since questionnaires were used to measure sedentary behavior, the data could be subjected to social desirability. However, only by using questionnaires, it is possible to include context-specific information on sedentary behavior. Second, the data included are relatively old and (the access to) screen devices (i.e., smartphones, tablets) and behaviors have changed since the data collection. Therefore, the interpretation of the current results might not be applicable for these new (mobile) screen devices. Future studies should include data collection regarding the use of smartphones and tablets in combination with the amount of TV viewing and computer time. Third, children were asked about the amount of time per day they spent on playing games or using a computer. No distinction was however made between those two concepts, which means that we do not know whether the results found in this study are applicable for the separate concepts. Future studies should think about which specific sedentary activities to study and how this should be done. Fourth, sedentary time on a weekday and on an average day (including weekend days) can differ and the corresponding correlates may differ as well [38]. In the current manuscript, daily TV time was based on weekdays only (before and after school), while daily computer time was based on an average day (including week and weekend days). It is therefore possible that different time frames used for TV and computer time might have influenced associations with SES indicators.

## Conclusion

Parental education was the most important SES determinant of both TV and computer use in children. In addition, ethnicity was the strongest predictor of computer time. A lower parental education leads to an increase of screen time behavior over time and screen time starts already at a higher level. Future interventions should target this important at-risk group.

## Supporting information

S1 Dataset Dataset used in the manuscript. Fully anonymised dataset.
(SAV)

## References

[1] Sedentary Behaviour Research Network. Letter to the editor: standardized use of the terms "sedentary" and "sedentary behaviours". Applied Physiology, Nutrition, & Metabolism = Physiologie Appliquee, Nutrition et Metabolisme. 2012;37(3):540–2. Epub 2012/05/01. 10.1139/h2012-024 . 22540258

[2] de Rezende LF, Rodrigues Lopes M, Rey-Lopez JP, Matsudo VK, Luiz Odo C. Sedentary behavior and health outcomes: an overview of systematic reviews. PLoS ONE [Electronic Resource]. 2014;9(8):e105620 Epub 2014/08/22. 10.1371/journal.pone.0105620 . 25144686 PMC4140795

[3] Proper KI, Singh AS, van Mechelen W, Chinapaw MJ. Sedentary behaviors and health outcomes among adults: a systematic review of prospective studies. American Journal of Preventive Medicine. 2011;40(2):174–82. Epub 2011/01/18. 10.1016/j.amepre.2010.10.015 . 21238866

[4] Thorp AA, Owen N, Neuhaus M, Dunstan DW. Sedentary behaviors and subsequent health outcomes in adults a systematic review of longitudinal studies, 1996–2011. American Journal of Preventive Medicine. 2011;41(2):207–15. Epub 2011/07/20. 10.1016/j.amepre.2011.05.004 . 21767729

[5] van der Ploeg HP, Chey T, Korda RJ, Banks E, Bauman A. Sitting time and all-cause mortality risk in 222 497 Australian adults. Archives of Internal Medicine. 2012;172(6):494–500. Epub 2012/03/28. 10.1001/archinternmed.2011.2174 . 22450936

[6] Chinapaw M, Altenburg T, Brug J. Sedentary behaviour and health in children—evaluating the evidence. Preventive Medicine. 2015;70:1–2. Epub 2014/12/03. 10.1016/j.ypmed.2014.10.029 . 25449691

[7] Atkin AJ, Sharp SJ, Corder K, van Sluijs EM. Prevalence and correlates of screen time in youth: an international perspective. American Journal of Preventive Medicine. 2014;47(6):803–7. Epub 2014/09/23. 10.1016/j.amepre.2014.07.043 . 25241193

[8] Canadian Society for Exercise Physiology. Canadian 24-Hour Movement Guidelines for Children and Youth 2016 http://www.csep.ca/view.asp?x=696.

[9] American Acadamy of Pediatrics. Media Use in School-Aged Children and Adolescents 2016 http://pediatrics.aappublications.org/content/early/2016/10/19/peds.2016-2592. 10.1542/peds.2016-2592 27940794

[10] Carson V, Hunter S, Kuzik N, Gray CE, Poitras VJ, Chaput JP, et al Systematic review of sedentary behaviour and health indicators in school-aged children and youth: an update. Appl Physiol Nutr Metab. 2016;41(6 Suppl 3):S240–65. Epub 2016/06/17. 10.1139/apnm-2015-0630 . 27306432

[11] Hardy LL, Mihrshahi S, Bellew W, Bauman A, Ding D. Children’s adherence to health behavior recommendations associated with reducing risk of non-communicable disease. Prev Med Rep. 2017;8:279–85. Epub 2017/12/20. 10.1016/j.pmedr.2017.10.006 . 29255663 PMC5723372

[12] Roman-Vinas B, Chaput JP, Katzmarzyk PT, Fogelholm M, Lambert EV, Maher C, et al Proportion of children meeting recommendations for 24-hour movement guidelines and associations with adiposity in a 12-country study. Int J Behav Nutr Phys Act. 2016;13(1):123 Epub 2016/11/27. 10.1186/s12966-016-0449-8 . 27887654 PMC5123420

[13] Pate RR, Mitchell JA, Byun W, Dowda M. Sedentary behaviour in youth. Br J Sports Med. 2011;45(11):906–13. Epub 2011/08/13. 10.1136/bjsports-2011-090192 . 21836174

[14] Salmon J, Tremblay MS, Marshall SJ, Hume C. Health risks, correlates, and interventions to reduce sedentary behavior in young people. American Journal of Preventive Medicine. 2011;41(2):197–206. Epub 2011/07/20. 10.1016/j.amepre.2011.05.001 . 21767728

[15] Mielke GI, Brown WJ, Nunes BP, Silva IC, Hallal PC. Socioeconomic Correlates of Sedentary Behavior in Adolescents: Systematic Review and Meta-Analysis. Sports Medicine. 2017;47(1):61–75. Epub 2016/06/05. 10.1007/s40279-016-0555-4 scholarship from the Coordinator for the Improvement of Higher Education Personnel (CAPES), Brazil. This work was supported by a partnership between CAPES, Federal University of Pelotas, Brazil, and the School of Human Movement and Nutrition Sciences, University of Queensland, Australia, that enabled international collaborative work to be undertaken. Conflict of interest Gregore Mielke, Wendy Brown, Bruno Nunes, Inacio Silva, and Pedro Hallal have no conflicts of interest relevant to the content of this review. 27260683 PMC5215067

[16] Pulsford RM, Griew P, Page AS, Cooper AR, Hillsdon MM. Socioeconomic position and childhood sedentary time: evidence from the PEACH project. Int J Behav Nutr Phys Act. 2013;10:105 Epub 2013/09/07. 10.1186/1479-5868-10-105 . 24007492 PMC3844440

[17] Coombs N, Shelton N, Rowlands A, Stamatakis E. Children’s and adolescents’ sedentary behaviour in relation to socioeconomic position. Journal of Epidemiology & Community Health. 2013;67(10):868–74. Epub 2013/07/16. 10.1136/jech-2013-202609 . 23851152 PMC3835391

[18] Babey SH, Hastert TA, Wolstein J. Adolescent sedentary behaviors: correlates differ for television viewing and computer use. J Adolesc Health. 2013;52(1):70–6. Epub 2012/12/25. 10.1016/j.jadohealth.2012.05.001 . 23260837 PMC3786734

[19] Brodersen NH, Steptoe A, Boniface DR, Wardle J. Trends in physical activity and sedentary behaviour in adolescence: ethnic and socioeconomic differences. Br J Sports Med. 2007;41(3):140–4. Epub 2006/12/21. 10.1136/bjsm.2006.031138 . 17178773 PMC2465219

[20] Atkin AJ, Corder K, Ekelund U, Wijndaele K, Griffin SJ, van Sluijs EM. Determinants of change in children’s sedentary time. PLoS ONE [Electronic Resource]. 2013;8(6):e67627 Epub 2013/07/11. 10.1371/journal.pone.0067627 . 23840753 PMC3695900

[21] Janssen X, Basterfield L, Parkinson KN, Pearce M, Reilly JK, Adamson AJ, et al Determinants of changes in sedentary time and breaks in sedentary time among 9 and 12 year old children. Prev Med Rep. 2015;2:880–5. Epub 2016/02/05. 10.1016/j.pmedr.2015.10.007 . 26844164 PMC4697128

[22] Gorely T, Marshall SJ, Biddle SJ. Couch kids: correlates of television viewing among youth. Int J Behav Med. 2004;11(3):152–63. Epub 2004/10/22. 10.1207/s15327558ijbm1103_4 . 15496343

[23] Van Der Horst K, Paw MJ, Twisk JW, Van Mechelen W. A brief review on correlates of physical activity and sedentariness in youth. Medicine & Science in Sports & Exercise. 2007;39(8):1241–50. Epub 2007/09/01. 10.1249/mss.0b013e318059bf35 . 17762356

[24] Caperchione CM, Kolt GS, Mummery WK. Physical activity in culturally and linguistically diverse migrant groups to Western society: a review of barriers, enablers and experiences. Sports Medicine. 2009;39(3):167–77. Epub 2009/03/18. 10.2165/00007256-200939030-00001 . 19290674

[25] Lakerveld J, van der Ploeg HP, Kroeze W, Ahrens W, Allais O, Andersen LF, et al Towards the integration and development of a cross-European research network and infrastructure: the DEterminants of DIet and Physical ACtivity (DEDIPAC) Knowledge Hub. Int J Behav Nutr Phys Act. 2014;11:143 Epub 2014/01/01. 10.1186/s12966-014-0143-7 . 25731079 PMC4245771

[26] Riddoch C, Edwards D, Page A, Froberg K, Anderssen SA, Wedderkopp N, et al The European Youth Heart Study—cardiovascular disease risk factors in children: rationale, aims, study design, and validation of methods. Journal of Physical Activity and Health. 2005;2(1):115–29.

[27] McMinn AM, van Sluijs EM, Wedderkopp N, Froberg K, Griffin SJ. Sociocultural correlates of physical activity in children and adolescents: findings from the Danish arm of the European Youth Heart study. Pediatr Exerc Sci. 2008;20(3):319–32. Epub 2008/08/21. . 18714121 10.1123/pes.20.3.319

[28] Brage S, Wedderkopp N, Ekelund U, Franks PW, Wareham NJ, Andersen LB, et al Objectively measured physical activity correlates with indices of insulin resistance in Danish children. The European Youth Heart Study (EYHS). International Journal of Obesity & Related Metabolic Disorders: Journal of the International Association for the Study of Obesity. 2004;28(11):1503–8. Epub 2004/08/25. 10.1038/sj.ijo.0802772 . 15326467

[29] Verbeke G, Molenberghs G. Linear Mixed Models for Longitudinal Data. New York: Springer-Verlag; 2000.

[30] Bates D, Maechler M, B B., Walker S, Bojesen Christensen RH, Singmann H, et al lme4: Linear mixed-effects models using Eigen and S4. R Package version 1.1–7 2014 http://CRAN.R-project.org/package=lme4.

[31] Christensen R. Analysis of ordinal data with cumulative link models—estimation with the R-package ordinal 2015. https://cran.r-project.org/web/packages/ordinal/vignettes/clm_intro.pdf.

[32] Gebremariam MK, Altenburg TM, Lakerveld J, Andersen LF, Stronks K, Chinapaw MJ, et al Associations between socioeconomic position and correlates of sedentary behaviour among youth: a systematic review. Obes Rev. 2015;16(11):988–1000. Epub 2015/09/01. 10.1111/obr.12314 . 26317685

[33] Ventura AK, Birch LL. Does parenting affect children’s eating and weight status? Int J Behav Nutr Phys Act. 2008;5:15 Epub 2008/03/19. 10.1186/1479-5868-5-15 . 18346282 PMC2276506

[34] Gebremariam MK, Altenburg TM, Lakerveld J, Andersen LF, Stronks K, Chinapaw MJ, et al Associations between socioeconomic position and correlates of sedentary behaviour among youth: a systematic review. Obesity Reviews. 2015;16(11):988–1000. 10.1111/obr.12314 26317685

[35] OECD. Better Life Index Denmark 2015. https://www.oecdbetterlifeindex.org/countries/denmark/.

[36] Brug J, Uijtdewilligen L, van Stralen MM, Singh AS, Chin AMJ, De Bourdeaudhuij I, et al Differences in beliefs and home environments regarding energy balance behaviors according to parental education and ethnicity among schoolchildren in Europe: the ENERGY cross sectional study. BMC Public Health. 2014;14:610 Epub 2014/06/18. 10.1186/1471-2458-14-610 . 24934085 PMC4067068

[37] Lowry R, Wechsler H, Galuska DA, Fulton JE, Kann L. Television viewing and its associations with overweight, sedentary lifestyle, and insufficient consumption of fruits and vegetables among US high school students: differences by race, ethnicity, and gender. J Sch Health. 2002;72(10):413–21. Epub 2003/03/06. . 12617028 10.1111/j.1746-1561.2002.tb03551.x

[38] Atkin AJ, Corder K, Ekelund U, Wijndaele K, Griffin SJ, van Sluijs EM. Determinants of change in children’s sedentary time. PLoS One. 2013;8(6):e67627 Epub 2013/07/11. 10.1371/journal.pone.0067627 . 23840753 PMC3695900

